# Direct Glutamatergic Signaling From Midbrain Dopaminergic Neurons Onto Pyramidal Prefrontal Cortex Neurons

**DOI:** 10.3389/fncir.2018.00070

**Published:** 2018-08-29

**Authors:** José Luis Pérez-López, Rubén Contreras-López, Josué O. Ramírez-Jarquín, Fatuel Tecuapetla

**Affiliations:** Instituto de Fisiología Celular (IFC), Neuropatología Molecular, Universidad Nacional Autónoma de México, Mexico City, Mexico

**Keywords:** co-release, dopamine, VTA, glutamate, prefrontal cortex

## Abstract

The dopaminergic neurons of the ventral tegmental area (VTA) have been identified with the ability to co-release dopamine and glutamate. This ability was first documented in the nucleus accumbens but showed to be absent in the dorsal striatum. Recently the ability to release glutamate from a subpopulation of the VTA dopaminergic neurons has been shown to control the prefrontal cortex (PFC) excitation through the exclusive innervation of GABAergic fast spiking interneurons. Here, using an optogenetic approach, we expand this view by presenting that the VTA dopaminergic neurons do not only innervate interneurons but also pyramidal PFC neurons. This finding opens the range of possibilities for the VTA dopaminergic neurons to modulate the activity of PFC.

## Introduction

The first data documenting the co-release of dopamine and glutamate by dopaminergic neurons dates back to 1998, when it was shown in cell cultures that dopaminergic neurons from the midbrain had the ability to release glutamate at their axonal terminals (Sulzer et al., 1998; Joyce and Rayport, 2000). Later on, the discussion of whether the co-release of glutamate by ventral tegmental area (VTA) dopaminergic neurons occurred *in vivo* was supported by VTA extracellular stimulation in brain slices and *in vivo* (Chuhma et al., 2004; Lavin et al., 2005), and by the description of VTA dopaminergic neurons containing the vesicular glutamate transporter type 2 (VGluT2; Dal Bo et al., 2008; Mendez et al., 2008). However, due to the nature of extracellular stimulation (it can activate fibers of passage), the selective activation of the VTA dopaminergic axons only came from the optogenetic activation of VTA axons expressing Channelrhodopsin-2 (ChR2; Stuber et al., 2010; Tecuapetla et al., 2010). In these experiments photo-activation of VTA dopaminergic axons in the nucleus accumbens evoked the release of glutamate in adult animals. Soon after, it was demonstrated that the selective deletion of VGluT2 in dopaminergic neurons eliminated the ability of VTA dopaminergic neurons to release glutamate, showing that the VGluT2 participated in the release of glutamate from dopaminergic cells (Fortin et al., 2012; Hnasko et al., 2012). Surprisingly, when similar optogenetic experiments were performed to evaluate whether the dopaminergic neurons also released glutamate in the dorsal striatum, this release was not observed (Stuber et al., 2010). This last finding provided evidence that not all post-synaptic targets of the dopaminergic neurons receive the co-release of dopamine-glutamate. Consequently, current research has focused on investigating whether dopaminergic axons co-release glutamate at their different targets (Kabanova et al., 2015; Ellwood et al., 2017; Mingote et al., 2017). Specific to our research, the first studies documenting the possibility that the VTA axons may release glutamate on prefrontal cortex (PFC) neurons were done by extracellular electrical stimulation in the VTA while recording PFC neurons (Mercuri et al., 1985; Lavin et al., 2005). Particularly important for our research, Lavin et al. (2005) presented a series of experiments strongly arguing in favor of the VTA providing a glutamatergic signal on PFC neurons, but due to the nature of their extracellular stimulation (it can activate fibers of passage or the VTA glutamatergic neurons; Morales and Root, 2014), no definitive answer was provided as to whether VTA dopaminergic neurons could directly release glutamate on PFC neurons. Later on, new studies searching for the co-release of glutamate by dopaminergic axons in the PFC have shown two opposite results. One study showed that dopaminergic axons do not co-release glutamate in the PFC (Mingote et al., 2015), whereas other works strongly suggested not only co-release of glutamate in PFC (Kabanova et al., 2015; Ellwood et al., 2017), but that these axons specifically innervate GABAergic fast-spiking interneurons (Kabanova et al., 2015) suggesting a specific control of the activity in PFC through the VTA→PFC(interneurons)→PFC(projection-neurons) circuit. However, the wide distribution of the dopaminergic cells from the VTA projecting to the PFC (Morales and Root, 2014) suggested an heterogeneous innervation onto the different PFC cell types. Therefore, with the aim to update these views, we performed whole cell recordings from neurons in the PFC while photo-activating the incoming VTA axons to ask if their release of glutamate was exclusive to control the cortical interneurons or they could also directly release glutamate onto the glutamatergic PFC neurons.

## Materials and Methods

### Animals

All procedures were approved by the Institutional Committee for the Care and Use of Laboratory Animals of the Cell Physiology Institute, National Autonomous University of México (CICUAL N° FTA91-16) and the National Norm for the use of Animals (NOM-062-ZOO-1999). Transgenic animals used in the experiments resulted from the backcrossing of TH-Cre mice (Tyrosine Hydroxylase, FI12 line, kindly donated by Professor Dr. Rui M. Costa, from the Champalimaud Center for the Unknown) into Black C57BL/6J for at least six generations. Male and female TH-Cre mice of 2–3 months of age were obtained from our breeding colony in our institutional animal facility (the TH-Cre line was conserved in heterozygosity) and were housed under a 12 h light/dark cycle (lights on at 6:00 am) with *ad libitum* access to food and water before experiments.

### Stereotaxic Virus Injections

To perform surgeries, animals were anesthetized using a mix of oxygen (1 liter/min) and 1% isoflurane (1%–2% for interventional procedures). After anesthesia and aligning of the animal’s head on the stereotactic apparatus, using an arm with 10 μm resolution (model 961, Kopf instruments), each animal was unilaterally injected using a glass pipette (>25 μm and <50 μm) with 500 nL of viral stock solution (AAV2.1-Ef1a-DIO-hChR2-(H134R)-eYFP titer 1 × 10^12^; Penn vector core, UPENN), using a nanoject-II (Drummond Scientific) programed to deliver 4.6 nanoliters by pressure each 5 s (23.6 nL/s rate) into the VTA. The coordinates for the injection with respect to Bregma were: anteroposterior −3.0 mm, mediolateral ±0.4 mm and dorsoventral −4.4 mm using as reference the Mouse Atlas (Paxinos and Franklin, 2004). The withdrawal of the pipette was done after 15–20 min to allow diffusion of the virus, the skin of the animals was subsequently sutured, and full recovery of each animal was monitored.

### *Ex vivo* Brain Slices and Data Acquisition

After allowing 10–14 days for ChR2 expression, the animals were deeply anesthetized with ketamine (120 mg/kg, i.p.; Anesket) and xylazine (30 mg/kg, i.p.; Bayer) and perfused transcardially with an ice-cold perfusion solution containing (in mM): 60 NaCl, 100 sucrose, 20 D-glucose 2.5 KCl, 5 MgCl_2_, 1.25 NaH_2_PO_4_, 26 NaHCO_3_, 1 CaCl_2_ (pH 7.3), saturated with 95/5% O_2_/CO_2_.

**Figure 1:**
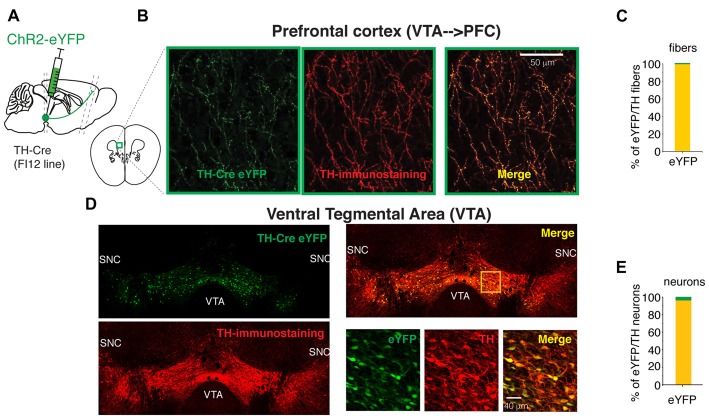
The FI12:TH-Cre mouse line is specific to targeting the midbrain dopaminergic neurons. **(A)** Diagram of a sagittal mouse slice depicting the site of viral injection to express either eYFP or Channelrhodopsin-2 (ChR2)-eYFP. **(B)** Photomicrographs of maximum intensity projection from a Z-stack of TH-Cre eYFP expressing fibers in the prefrontal cortex (PFC; prelimbic, PrL cortex). These projections arise from the cell bodies labeled in the ventral tegmental area (VTA), the z-stack is from 10 photomicrographs taken every 2 microns. **(C)** Specificity to express eYFP in the TH-expressing PFC fibers. **(D)** Photomicrographs of the injected site in the VTA of a mouse FI12:TH-Cre with AAV2.1 DIO-eYFP. The three bottom right panels show the magnification of the yellow square in the upper right panel. **(E)** Specificity to express eYFP in VTA TH Cre/TH+ neurons. For this figure we quantified all cells per slice from the VTA (see “Materials and Methods” section) from three animals.

Coronal or angled slices (250–300 microns) were obtained at the PFC level (angled slices had a 30–40 degree-angle from the coronal axis as illustrated in Figure 1A) using a vibratome (3000 Ted pella), slices were then transferred to a storage chamber with oxygenated artificial cerebrospinal fluid (aCSF) containing (in mM): 125 NaCl, 3 KCl, 1.3 MgCl_2_, 2.6 CaCl_2_, 26 NaHCO_3_, 1.25 NaH_2_PO_4_, 10 glucose, 3 sodium pyruvate (pH 7.3, 310 mOsmol/L) and allowed for 1 h recovery at room temperature. Single slices were transferred to a submerged recording chamber and superfused continuously with oxygenated aCSF (3–5 mL/min). Whole-cell recordings were performed on PFC neurons at room temperature, with micropipettes made from borosilicate glass (Harvard apparatus 30-0057) and fire polished for DC resistances of 6–10 MΩ. Internal solution was (in mM): 10 NaCl, 10 HEPES, 10 EGTA/KOH, 120 KMeSO_3_, 2 MgCl_2_, 1 CaCl_2_, 2.4 Na^+^-ATP, 1.2 Na^+^-GTP and 3.3 biocytin (pH 7.2, 290 mOsmol/L). Neurons were visualized using an infrared filter with an upright microscope (Scientifica electrophysiology) and a digital camera (Evolution VF FAST mono 12-bit 32-0103B124). Whole cell recordings were acquired through a PC-501A amplifier (Warner Instrument Corp.) and data was digitized through a NIDAQ (CB 68LP, National Instruments) and Im-Patch (open access software designed in LabView).

### Optogenetic Activation of VTA ChR2:TH-Cre Axons Into the PFC

Once intracellular access was obtained (holding potential −70 mV in voltage clamp or resting membrane potential in current clamp), bath application of 4-aminopiridine (40 μM, A0152 Sigma-Aldrich) was used to improve activation of ChR2 expressing axons (Petreanu et al., 2009), synaptic responsivity (Flores-Hernández et al., 1994), and the detection of the postsynatic responses (Kabanova et al., 2015). For optical stimulation of VTA:TH-Cre ChR2-eYFP axons in the PFC, 20 ms pulses of blue light (otherwise specified; 470 nm; 3 mW; CoolLED pE-100) were delivered through the same objective used to visualize the cells (40×). Once a postsynaptic response was detected, traces were recorded every 10 s. The experimental protocol continued by first varying the holding potential to obtain the reversal potential of the postsynaptic response. Then, glutamatergic (CNQX; 10 μM, C239 Sigma-Aldrich) or the GABA-A (SR-95531; 10 μM S106 Sigma-Aldrich) antagonists were added to the perfusate. At least 30 traces were acquired in the control and the antagonist conditions.

### VTA and PFC Immunostaining to Identify Cell Bodies and Fibers

The brains from animals injected to express eYFP in the TH-Cre VTA neurons were obtained by first deeply anesthetizing and transcardially perfusing the animals with PBS 0.1 M and 4% paraformaldehyde (PFA). After overnight post-fixation in 4% PFA, brains were washed in PBS five times, and coronal or angled sections were obtained from the VTA or PFC in PBS 1% (50 μm slices) using a vibratome (3000 Ted pella). For immunostaining, the tissue sections were permeabilized with 0.3% Triton X-100 in PBS for 10 min at room temperature. After a blocking step (incubation for 40 min, at room temperature with 10% FBS, 0.3% Triton X-100:PBS), and depending on the experiment, sections were incubated with a primary antibody: 12 h at room temperature when using the polyclonal antibody against TH, 1:500 dilution (AB152, Merck Millipore) or 18–24 h when using the monoclonal antibody against CaMKII, 1:1,000 dilution (EP 1819Y, Abcam). Next, after five washes with PBS, a secondary antibody conjugated with the Cy3 or alexa 594 in the case of the TH primary antibody (711-165-152, Jackson ImmunoResearch) or a secondary antibody coupled with Alexa 647 in the case of CaMKII (711-605-152 Jackson ImmunoResearch) were incubated for 2 h in a 1:1,000 dilution. When necessary, DNA was counterstained with DAPI or Hoechst. Slices containing the PFC and the VTA were mounted and images were acquired with an LSM710 laser-scanning confocal microscope (Carl Zeiss). 20× magnification Z-stacks of two channels, one to detect eYFP and one for TH-Cy3 or TH-alexa 594 (excitation lasers were 488 and 543 for eYFP and Cy3 or alexa 594, emission 507–560 nm and 585–648 nm, respectively; 424 × 424 × 20 microns, 2 microns interslice). One slice every three in the VTA area (covering from −2.8 mm to −4.0 mm anteroposterior from Bregma) was selected for the quantification of eYFP+/TH+ cells. Quantification of eYFP-TH+ fibers in the PFC was done through Z-stacks taken from a randomly generated grid inside the limits of the recording areas (prelimbic, PrL and infralimbic, IL PFC; slice anteroposterior to Bregma 1.54 mm). The Z-stacks were imported to ImageJ, then a maximum projection image was obtained and fibers were quantified if they crossed a randomly positioned grid of 20 microns through more than two horizontal lines (Grider et al., 2006).

Recorded cells were processed for anatomy through a reaction to detect Biocytin (1:100, Streptavidin-Cy3, 434315 Thermo Fisher Scientific) and further processed to detect CaMKII. Once samples were mounted, a Z-stack of the area of interest was acquired, images were imported to ImageJ and estimations were performed.

### Statistical Analysis

All data is presented as mean ± SEM, unless otherwise specified in the text. The significance level used was *p* < 0.05. For non-paired comparisons Mann-Whitney U test was performed, for percentage distributions comparison Chi square test were used and for paired samples Wilcoxon *t*-test was used. All statistic analyses were performed using Graph Pad and MATLAB.

### Data Availability

All data is available upon contact with the corresponding author.

## Results

To elucidate whether the dopaminergic neurons from the VTA release glutamate in the PFC, the specific activation of their axons in PFC was required. To achieve this specific activation of the VTA dopaminergic axons, the mouse line FI12:TH-Cre, that expresses the enzyme Cre recombinase under the promoter for tyrosine hydroxylase was used (Gong et al., 2007; da Silva et al., 2018). Figure 1 shows photomicrographs of eYFP-expressing cell bodies in the VTA (Figure 1D) and their axonal projections to the PFC (Figures 1A,B).

The quantification of eYFP+/TH+ fibers and cell bodies in randomly selected Z-stacks of the PFC (two quadrants of 150 microns from a randomly positioned grid on PFC were used to estimate the eYFP-fibers) and all cells transfected in the VTA (*n* = 3 animals) showed the specificity of the FI12:TH-Cre for targeting the cell bodies of VTA dopaminergic neurons (defined as a TH+ cell) was 96.32% (Figure 1E). Furthermore, the specificity of dopaminergic fibers in the PFC originating from VTA neurons was 99% (Figure 1C). This specificity allowed us to use the FI12:TH-Cre mouse line to express proteins of interest in VTA neurons and their fibers reaching the PFC.

### Heterogeneous Postsynaptic Currents Are Evoked in Prefrontal Cortex by the Activation of the VTA-ChR2:TH-Cre Axons

**Figure 2:**
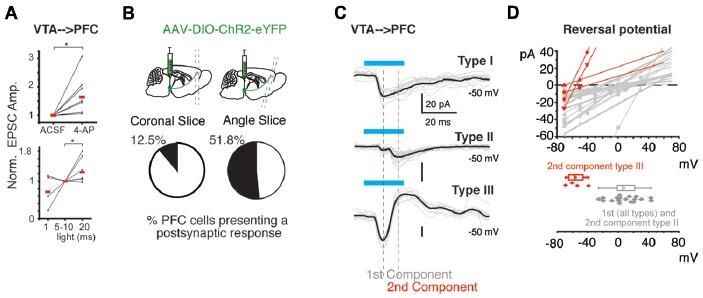
Heterogeneity of postsynaptic currents (PSCs) evoked in PFC by the activation of VTA-ChR2 axons. **(A)** Upper panel, normalized amplitude of PSCs recorded before and in the presence of 4-aminopyridine (4-AP; 40 μM), lower panel, normalized amplitude of PSCs in response to different length of light pulses. **(B)** Representative diagrams of sagittal brain slices depicting the angled cut to obtain PFC brain slices. Bottom pie charts: probability to evoke postsynaptic responses by the photo-activation of VTA:TH-Cre-ChR2-eYFP axons in PFC. **(C)** Two categories of postsynaptic responses were detected: Category A: presents only one component (Type 1: only inward current). Category B: presents two components (Type II: present two inward currents; Type III presents two components one inward current followed by an outward current). Blue lines depict the light stimulus to activate ChR2. The black and red vertical lines depict the points where the first and second component amplitude was measured. For all types of connections, a smaller light pulse (1–10 ms) did not change the number of postsynaptic components but the amplitude was smaller (see “Heterogeneous Postsynaptic Currents Are Evoked in Prefrontal Cortex by the Activation of the VTA-ChR2:TH-Cre Axons” section). **(D)** Upper panel, lines are linear fits of the experimental data (points) per cell varying the holding potential from −70 mV to +20 mV. Bottom panel: distribution of the reversal potential calculated from the data above. *p < 0.05 Wilcoxon test.

In order to answer whether the selective activation of the VTA TH-Cre axons release glutamate in the PFC, we expressed the light sensitive protein ChR2 (Boyden et al., 2005) into the TH-Cre neurons of the VTA, and 10–14 days later PFC brain slices were obtained. Using these brain slices, we asked whether performing whole cell recording from PFC neurons and light activating the VTA TH-Cre axons expressing ChR2-eYFP (VTA-ChR2:TH-Cre axons) could evoke postsynaptic currents (PSCs), by light activating the VTA-ChR2:TH-Cre axons in the PFC, as previously reported (Kabanova et al., 2015; Ellwood et al., 2017). Following these procedures and in an attempt to improve the activation of ChR2 expressing axons (Petreanu et al., 2009), the synaptic responsivity (Flores-Hernández et al., 1994) and the detection of the postsynatic responses (Kabanova et al., 2015) we added 4-aminopyridine (4-AP) to the extracellular recording solution. In agreement with the literature, 4-AP facilitated the amplitude of PSC recorded in PFC neurons in response to light activation of the VTA-ChR2:TH-Cre axons (the normalized amplitude increased in average to 1.6 times, seven cells, from seven animals; *p* < 0.05 Wilcoxon test; Figure 2A, top panel; amplitude in ASCF 19 ± 4 pA vs. 29 ± 8 in 4-AP *p* < 0.05 Wilcoxon test; rise and decay time of 2.7 ± 0.3 and 13 ± 1.9 ms respectively). Similarly, the delivery of 20 vs. 10 or 1 ms pulses of light facilitated the amplitude to 1.3 times (six cells from six different animals, *p* < 0.05 Wilcoxon test, Figure 2A, bottom panel). Four of these cells had a single component using 1 or 10 ms pulses of light, and no additional components appeared using 20 ms pulses. Strikingly, it was also observed that the probability to detect postsynaptic responses was dependent on the type of brain slices used. Using brain slices with an angle (see “Materials and Methods” section) yielded a higher probability to detect postsynaptic responses (12.5% PSC detected in coronal slices; 3 out of 24 recorded neurons from 12 slices from 9 animals; vs. 51.8% in angled slices; 41 out of 79 recorded neurons from 35 slices from 29 animals; *p* < 0.05; χ^2^ test; Figure 2B).

These PSCs were heterogeneous and grouped into two categories: Type I, which have a single component (20 out of 26 cells; 20 slices, 12 animals) and a second category: Type II and III, which have two components (Type II showed a second component that was an inward current at −50 mV; 4 out of 26 cells; four slices from four animals) and Type III showed a second component that was an outward current at −50 mV (4 out of 24 cells; four slices from four animals; Figure 2C). Most of these recordings were performed in angled slices (see Table 1). By performing a protocol to infer the reversal potential of these PSCs (varying the holding potential from −70 mV to +20 mV), and measuring the peak amplitude for the first and the second postsynaptic components, it was observed that the first postsynaptic component from all recordings has a higher reversal potential than the second component for Type III (mean of the first component 9 ± 4.7 mV vs. −55 ± 6.4 mV for the second component (Type III); *p* < 0.05; Mann Whitney U test; Figure 2D; reversal potential for Type II was 0 mV). These measurements of the first component and the second component of Type III resembles the theoretical reversal potential for PSCs mediated by glutamate and GABA, respectively.

**Table 1:** Postsynaptic currents (PSCs) evoked by the activation of VTA-ChR2:TH-Cre axons onto prefrontal cortex (PFC) neurons.

	Type	Postsynaptic current (s; −50 mV)	*n* total	*n* per slice type	
				Coronal	Angled
Category one	I	Inward ↓	*n* = 20	*n* = 2	*n* = 18
Category two	II	Inward-inward ↓↓	*n* = 4	*n* = 1	*n* = 3
	III	Inward-outward ↓↑	*n* = 4		*n* = 4

### The Postsynaptic Responses Evoked in the Prefrontal Cortex by the Photo-Activation of VTA-ChR2:TH-Cre Axons Are Mediated by Glutamate

**Figure 3:**
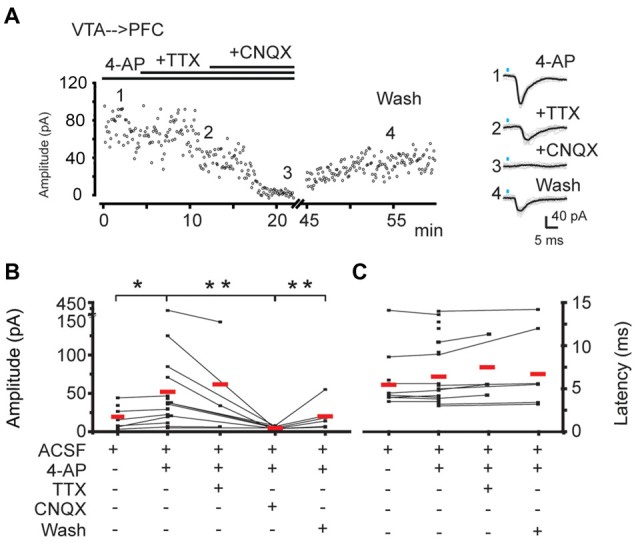
The postsynaptic responses evoked in PFC by the photo-activation of VTA-ChR2:TH-Cre axons are mediated by glutamate. **(A)** Left panel, time course for the peak amplitude of individual traces evoked by the photo-activation of VTA-ChR2:TH-Cre axons in PFC. Right panel, average of at least 20 traces (black traces) corresponding to the numbers depicting the time course. **(B)** Individual mean amplitude of every experiment in the different presented conditions (black points). Lines represent the experiments in which the same cell undergoes different conditions. Red lines are the mean of each condition. **(C)** Latency of the responses for the amplitudes measured in **(B)**. *Wilcoxon paired test *p* < 0.05; **Wilcoxon paired test and Mann Whitney U test *p* < 0.05.

To evaluate if the postsynaptic responses recorded on PFC neurons by the photo-activation of the VTA-ChR2:TH-Cre axons was mediated by glutamate, in nine of the postsynaptic responses detected, the AMPA glutamate receptor antagonist (CNQX 10 μM; nine slices from nine animals) was added to the bath perfusion. CNQX decreased the mean amplitude of the postsynaptic response to 9% of its original amplitude (control amplitude 52 ± 19 pA vs. 5 ± 0.7 pA in the presence of CNQX; *p* < 0.05; Mann Whitney U test; partial wash 20 ± 7 pA, Figure 3A and fourth column in Figure 3B). This suggests that the main neurotransmitter responsible for these postsynaptic signals is glutamate. Note that the latency for the mean postsynaptic response was not different before vs. during the partial wash of CNQX (control latency 7 ± 0.9 ms, latency after CNQX wash 7 ± 1.0 ms, *p* > 0.05; Mann Whitney U test, Figure 3C). Additionally, in three cells (out of 12, six slices, six animals) we achieved the recordings of postsynaptic responses in the presence of 4-AP+TTX (1 μM) arguing in favor of the VTA→PFC connection as monosynaptic (Figure 3A and third column in Figure 3B). Interestingly in these three cases the latency to detect the postsynaptic response was increased (see the right panel in Figure 3A), a feature of the EPSC evoked by light activation of ChR2 axons (Holloway et al., 2013), Two of these three cells were revealed as pyramidal neurons (further documented in “Photoactivation of VTA ChR2:TH-Cre Axons in the PFC Elicits Postsynaptic Currents Directly on Projection Neurons and Putative Interneurons” section).

### In 30% of the Cases Under 4-AP and the Optogenetic Activation of the VTA Axons Onto PFC Neurons, the Blockade of the GABA-A Receptors Enhanced the Postsynaptic Responses

**Figure 4:**
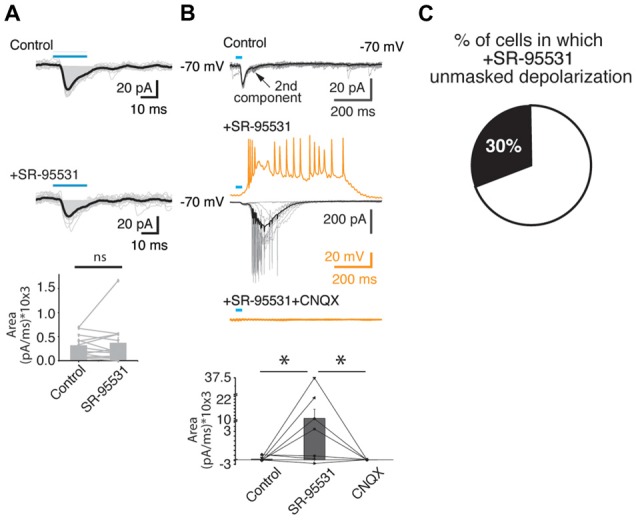
The blockade of the GABA-A receptors enhanced the glutamatergic PSCs evoked by the activation of VTA-ChR2 axons in PFC. **(A)** Upper two panels, representative example of a Type I postsynaptic response in control condition, and in the presence of the antagonist of GABA-A receptors (SR-95531; middle panel). The lower panel shows the area under the curve for a group of cells (13 cells, from 13 slices from 13 animals). **(B)** Representative recordings from a Type III postsynaptic response in voltage (black/gray traces) and current clamp (orange traces) showing that the bath application of the GABA-A antagonist receptors enhanced an inward current (voltage clamp) or a depolarization (current clamp) that later on was blocked by the co-application of the AMPA receptors antagonist (CNQX). Note that when in voltage clamp (control condition) the holding potential is −70 mV, which shows both postsynaptic components as inward currents. The bottom panel shows the summary (area under the curve) for the Type II-III postsynaptic responses that undergo the blockade of the GABA-A and AMPA receptors. **(C)** The proportion of PFC neurons that presented the depolarization after SR-95531 application. *p < 0.05 Mann Whitney U test.

Given that the release of glutamate from a subpopulation of the VTA dopaminergic neurons in the PFC has been shown to exclusively impinge on PFC interneurons (Kabanova et al., 2015), and IPSCs mediated by VTA-induced feedforward activation of local PFC interneurons has been suggested (Lavin et al., 2005), we asked whether the blockage of GABA-A receptors could affect the postsynaptic responses detected. For this purpose, we bath applied the specific GABA-A antagonist, SR-95531, and evaluated the changes in the postsynaptic signals detected (Figure 4). Consistent with the idea that the first component of the postsynaptic response in PFC is mediated mainly by glutamate, the application of a GABA-A antagonist did not modulate this first component (control area under the curve = 309 ± 61 pA/ms vs. 361 ± 122 pA/ms; 13 cells from 13 slices from 10 animals; *p* > 0.05; Mann Whitney U test; Figure 4A). However, in agreement with the idea that some of the dopaminergic axons of the VTA directly innervate GABAergic interneurons in the PFC, (Lavin et al., 2005; Kabanova et al., 2015), in 30% of the cases (4 out of 13 cells, from 10 animals) where the GABA-A receptors were blocked, the second glutamatergic postsynaptic component increased dramatically. This effect can be seen in the upper three panels of Figure 4B (control, +SR-95531, +SR-95531+CNQX) of one cell recorded in voltage clamp (black traces) and in current clamp (orange traces). The panel at the bottom of the same figure shows that in all cases where the blockade of the GABA-A receptors was followed by the subsequent bath application of CNQX, in the presence of SR-95531 (independently of been connections Type II or III), it abolished completely the postsynaptic responses (area under the curve: control = 91 ± 142 pA/ms; SR-95531 = 10,146 ± 5,604 pA/ms; *p* < 0.05; Mann Whitney U; SR-95531 + CNQX 10 ± 10 pA/ms; six out of six cells tested from six animals).

In summary, the blockade of the glutamatergic transmission practically abolished the postsynaptic responses detected, and, in a subset of cells (30%), the blockade of the GABA-A receptors enhanced the PSCs that were eliminated when blocking the glutamatergic transmission (Figure 4C).

### Photoactivation of VTA ChR2:TH-Cre Axons in the PFC Elicits Postsynaptic Currents Directly on Projection Neurons and Putative Interneurons

**Figure 5:**
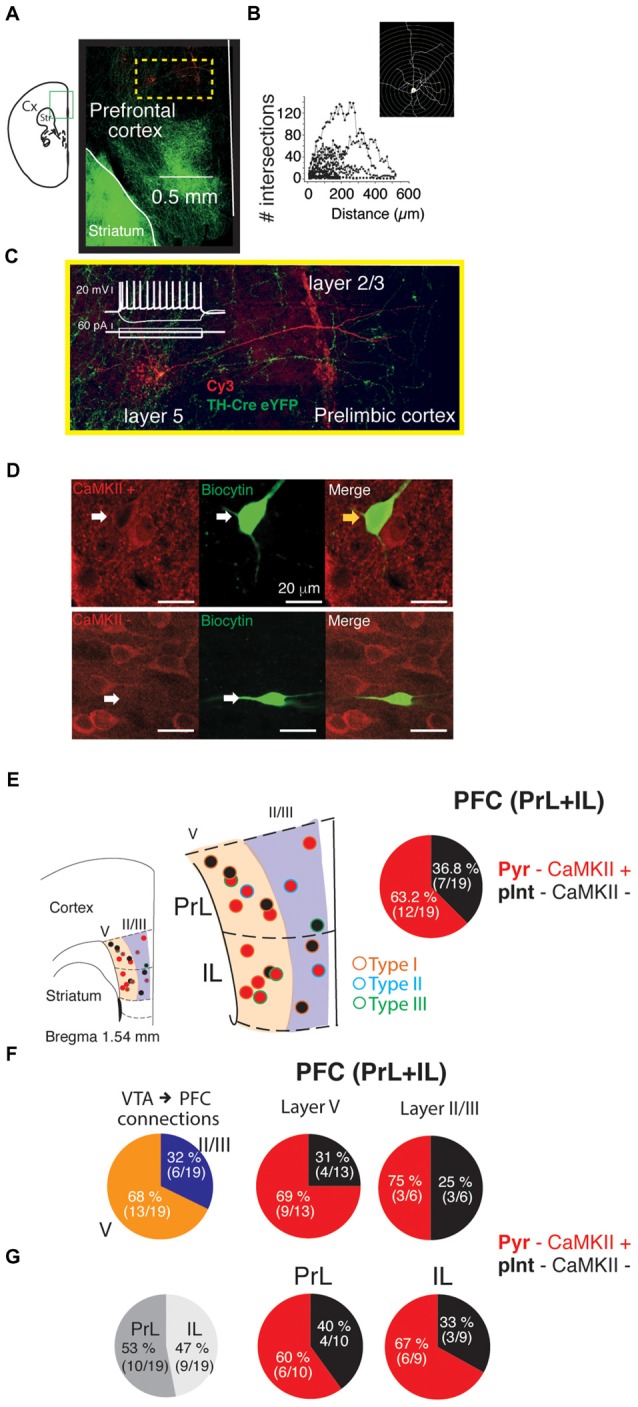
Proportion of calcium calmodulin type II (CaMKII)+ and CaMKII− PFC neurons that received the direct release of glutamate by the photo-activation of VTA-ChR2:TH-Cre axons. **(A,C)** Photomicrograph of a PFC neuron filled with biocytin labeled with Cy3. **(B)** Sholl analysis for 17 neurons recorded and processed as in **(A)**. **(C)** Magnification of the yellow square from **(A)** to better depict the neuron dendrites. Inset is the voltage responses (top) to square pulses of current (bottom). **(D)** Example of a CaMKII+ or CaMKII− neurons. **(E)** Left, diagram representing a section of a coronal brain slice to depict where the cells were recorded (red circles: CaMKII+ pyramidal neurons and black circles: CaMKII− putative interneurons). The panel in the middle represents a magnification of prelimbic (PrL) and infralimbic (IL) cortex representing by colors the layers where the cells were recorded. Right pie chart, proportion of pyramidal neurons vs. putative interneurons grouped as PFC neurons recorded.**(F)** Proportion of cells recorded by layer (left), and pyramidal neurons vs. putative interneurons recorded by layer (middle and right). **(G)** Proportion of pyramidal and putative-interneurons recorded in PrL or IL cortex.

To reveal the identity of PFC neurons that showed a glutamatergic EPSC from VTA ChR2:TH-Cre, recorded neurons were filled with biocytin and subsequently processed for morphological and immunohistochemical analysis (e.g., Figure 5A). Sholl analysis was performed on 17 recovered neurons. However, this analysis failed to reveal the identity of the cells (no clear distinction between pyramidal vs. interneurons could be reached; Figure 5B), likely due to an incomplete filling of the cells. Therefore, slices were resectioned and immunostained for calcium calmodulin Type II protein (CaMKII; a molecular marker used to differentiate glutamatergic neurons from interneurons in cortex (Pinto and Dan, 2015; Figure 5D). Thus, every cell showing positive label for CaMKII+ was designated as a pyramidal glutamatergic neuron and the CaMKII− as a putative interneuron. Following this procedure, the position and proportion of pyramidal neurons vs. putative interneurons was obtained as shown in Figure 5E; each dot corresponds to a recorded cell with a postsynaptic light-evoked response, superimposed on the average slice recorded (in red: CaMKII+: glutamatergic pyramidal neurons and in black CaMKII−: putative interneurons). This analysis showed that 12 out of the 19 identified neurons that received the direct glutamate release from the photo-activation of the VTA-ChR2:TH-Cre axons in the PFC were pyramidal neurons (63%). Three pyramidal and one putative interneuron received Type III connections, three pyramidal neurons presented connections Type II, and 19 Type I. Importantly from the five cells where the GABA-A antagonist enhanced the PSCs (subsequently blocked by CNQX; Figure 4), three were pyramidal and two putative interneurons. A comparison between neurons recorded by layers or recorded in PrL vs. IL cortex showed no difference in the proportion of CaMKII+ vs. CaMKII− recorded neurons (*p* > 0.05; χ^2^ test; Figures 5F,G).

In summary, based on our classification of the neurons that received direct release of glutamate in response to photo-activation of the VTA-ChR2:TH-Cre axons in the PFC, a similar proportion of pyramidal (CaMKII+) and putative interneurons (CaMKII−), received glutamate release.

## Discussion

Overall, the data presented here support the idea that the VTA dopaminergic neurons release glutamate on the PFC neurons. Notably, while our results support previous reports of glutamate release by VTA dopaminergic axons in the PFC (Mercuri et al., 1985; Lavin et al., 2005; Kabanova et al., 2015; Ellwood et al., 2017), our study specifically identified that this glutamate release occurs not only onto interneurons but also onto pyramidal neurons.

### Why Have Some Groups Documented the Release of Glutamate From VTA Axons in the PFC and Others Have Not?

The first attempts to document the capability of VTA axons to release glutamate on PFC neurons were by electrical (or iontophoretic) activation of the VTA while electro-physiologically recording in the PFC *in vivo*. These studies revealed postsynaptic responses that were blocked by glutamatergic antagonists and in some cases enhanced by GABA-A antagonist (Mercuri et al., 1985; Lavin et al., 2005). However, due to the nature of their VTA activation in those studies (as extracellular stimulation may activate fibers of passage or the VTA glutamatergic neurons that project to PFC; Morales and Root, 2014), no conclusive answer was provided to whether the VTA dopaminergic neurons directly released glutamate on PFC neurons. Latter with the use of transgenic mice, the answer to this question proposed two different views. In the first view, two groups documented glutamate co-release from VTA dopaminergic axons in the PFC using either a transgenic line that targets a specific subpopulation of dopaminergic neurons in the medial VTA (Kabanova et al., 2015) or a TH-Cre line (Ellwood et al., 2017), as in this study. In a second view, a different group using a DAT-IRES-Cre mouse line reported that such glutamate co-release did not exist (Mingote et al., 2015). This discrepancy raises the question of what may be the differences between these transgenic lines? The main difference could be that the TH-Cre and the DAT-Cre mouse lines target overlapping but not identical populations of VTA neurons (Björklund and Dunnett, 2007; Lammel et al., 2015; Stuber et al., 2015). These lines likely target different populations or compartments of the VTA (e.g., the medial vs. the lateral VTA), as these different compartments have been shown to differentially project to PFC (Morales and Root, 2014; Kabanova et al., 2015). Of crucial consideration is that when using transgenic lines to target specific populations of neurons of the midbrain, the specificity of each line may vary. This is especially true for the TH-Cre lines (Lammel et al., 2015), nonetheless it must be noted that the particular mouse line used in this study (FI12) has been documented for its specificity to target midbrain dopaminergic neurons (Gong et al., 2007; da Silva et al., 2018), and we further validated it in this study. By quantifying the cell bodies in the VTA and PFC axonal fibers originating in the VTA, we estimated a 96% and a 99% of specificity, respectively (Figure 1). This high level of specificity allowed us to support the idea that the release of glutamate in PFC evidenced by this study comes from a specific subset of VTA dopaminergic neurons in the FI12:TH-Cre line.

Intriguingly, a second possibility to explain why some groups have not detected the release of glutamate from the VTA dopaminergic neurons in the PFC may come from the different recording conditions in each study. Specifically we improved the reliability to detect the release of glutamate from VTA dopaminergic fibers into the PFC by using 4-AP in the extracellular solution (Petreanu et al., 2009; Kabanova et al., 2015), 20 ms pulses of light and an angled slice (Figures 2A,B). Presumably, and according to our data, this protocol improves the activation of the ChR2 expressing axons (Petreanu et al., 2009) the detection of the postsynatic responses (Kabanova et al., 2015) and better preserves incoming VTA axons in the angled slices, as the cell bodies from the VTA do not remain in the brain slices.

### Different Types of Postsynaptic Responses Due to the Activation of the VTA Dopaminergic Axons in PFC

Knock out animals with the deletion of the VGluT2 specifically from dopaminergic neurons, which removes their ability to co-release glutamate (Hnasko et al., 2012), show deficits in their motor control, their response to amphetamine or cocaine, and behavioral despair (Birgner et al., 2010; Alsio et al., 2011; Hnasko et al., 2012). Optogenetic manipulations of the dopaminergic VTA→PFC fibers (that release dopamine and glutamate) caused mice to maintain or deviate from previously learned cue–reward associations (Ellwood et al., 2017). The loss of mesocortical dopaminergic neurons that release glutamate in the PFC increases the perseverative behaviors and present alterations in impulsivity and attention (Kabanova et al., 2015). All these studies highlight the control that the VTA→PFC axons exert on PFC functions. However, to fully understand this control, we must first identify the full connectivity of this circuit. In 2015 Kabanova and colleagues (Kabanova et al., 2015) presented the first study of specific functional connectivity from VTA dopaminergic neurons that release glutamate on PFC neurons, pointing out a subpopulation of dopaminergic cells in the medial VTA that release glutamate exclusively on PFC interneurons. This finding raised the possibility that the VTA could exert control on the PFC projection neurons through GABAergic interneurons. Nevertheless, the heterogeneous distribution of the VTA fibers within the PFC (Figure 1B), data from the first author of this study during his bachelor thesis (Pérez-Lopéz, 2016), identifying that putative PFC pyramidal neurons receive glutamate release from VTA axons, and the possibility that a bigger population than the one described by Morales and Root (2014) and Kabanova et al. (2015) could innervate the PFC, kept the possibility of a more heterogeneous innervation from the dopaminergic VTA neurons in the PFC. Therefore, when investigating this possibility, we identified that most of the times that we were able to evoke postsynaptic responses in PFC, the neurons recorded were electrophysiologically identified as pyramidal-like neurons (see inset in Figure 5C white color). Nonetheless, since neither the sorting of the basic electrophysiology parameters in current clamp (input resistance, resting membrane potential and shape of action potentials; data not shown) nor the anatomical reconstruction (performing a Sholl analysis: Figure 5B), yielded a clear identification of the cells as pyramidal neurons vs. interneurons, an analysis of the recorded cells was performed to label for a the molecular marker of pyramidal neurons, CaMKII (Pinto and Dan, 2015; Figure 5D). The labeling of CaMKII showed that more than 60% of the recorded neurons were pyramidal neurons (12 out of 19). Additionally in a separated experiment the blockage of the GABA-A receptors, presented a strong depolarization linked to the optogenetic stimulation of VTA-axons in PFC (30% of the cases; Figure 4C), suggesting that as previously reported, some of the VTA dopaminergic axons release glutamate onto PFC GABAergic interneurons (Lavin et al., 2005; Kabanova et al., 2015; Figure 4B). This data highlights that around 70% of the VTA-TH axons release glutamate directly onto pyramidal PFC neurons. Notably, since our strategy to target VTA neurons did not differentiate between dopaminergic subpopulations (Björklund and Dunnett, 2007), an open question is whether the VTA-TH axons that innervate pyramidal PFC neurons come from a specific subpopulation in the VTA that directly drives the activation of PFC neurons (parallel to the one innervating the PFC interneurons).

### When Could the Co-release of Dopamine and Glutamate by VTA Axons in PFC Be Relevant?

Despite the fact that in our study we focused only on the capability of VTA axons in PFC to release glutamate, the ability of these same axons to release dopamine in the PFC has been previously documented (Garris et al., 1993; Hedou et al., 2001; Phillips et al., 2004; Lavin et al., 2005; Ellwood et al., 2017). Evidence from recording the local field potential in the PFC while stimulating the VTA showed a signal with three components, two fast components that correspond to the fast depolarization evoked by the release of glutamate triggered by VTA activation, and a third component of opposite amplitude that corresponds to an inhibitory postsynaptic response evoked by this same activation (Lavin et al., 2005). Importantly, at the level of EEG recordings, a switch to frequencies linked to the arousal state has been documented during electrically or optogenetically activation of the VTA (Solt et al., 2014; Taylor et al., 2016), this switch may come from the ability of VTA cells to co-release dopamine and glutamate on their different targets. Therefore, to answer the question of when it could be relevant that the VTA axons co-release dopamine and glutamate in the PFC two general ideas could be proposed. The first hypothesis proposes that the rapid release of glutamate from these axons may convey the fast salience of a stimulus, while the slower actions of dopamine may control the activity of PFC neurons on a slower scale (Lavin et al., 2005; Buchta et al., 2017). A second proposal is that the patterns of activation of VTA dopaminergic-glutamatergic axons in the PFC control whether mouse maintains or deviates from previously learned cue reward associations (Ellwood et al., 2017).

## General Conclusion

**Figure 6:**
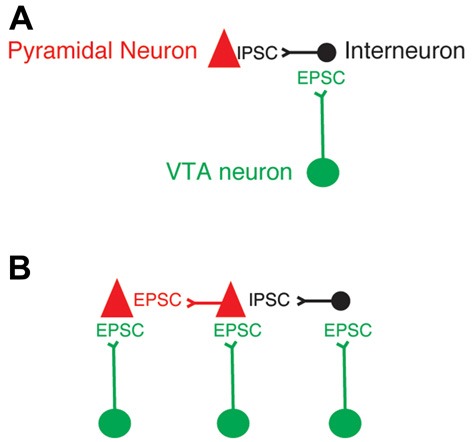
Updated model of VTA axons that release glutamate in PFC neurons. **(A)** Model of the exclusive innervation from VTA dopaminergic axons that release glutamate onto the GABAergic PFC interneurons. **(B)** Updated model based on the conclusion of this study.

This study showed that the axons from the VTA TH+ neurons that release glutamate on PFC directly innervate PFC pyramidal neurons. This finding expands the range of possibilities that VTA dopaminergic axons may have to modulate the spiking activity and functions of PFC neurons (Figure 6). Specific experiments evaluating the contribution of the release of glutamate from VTA axons onto pyramidal neurons vs. interneurons in PFC are necessary to fully visualize the functions of the co-release of glutamate from dopaminergic axons in the PFC.

## Author Contributions

JLP-L and FT designed and wrote the study. RC-L performed the 4-AP+TTX experiments. JOR-J provided the genotyping and technical support for the development of the study.

## Conflict of Interest Statement

The authors declare that the research was conducted in the absence of any commercial or financial relationships that could be construed as a potential conflict of interest.
